# Does an Intradermal Vaccination for Monkeypox Make Sense?

**DOI:** 10.1208/s12248-022-00754-6

**Published:** 2022-10-04

**Authors:** Randall J. Mrsny

**Affiliations:** Department of Life Sciences and Centre for Technology Innovation, University of Bath, Bath, BA2 7AY UK

**Keywords:** FDA guidance, Intradermal, Monkeypox, Vaccination

## Abstract

Mankind has recently had to deal a series of virus-mediated pandemics, resulting in extensive morbidity and mortality rates that have severely strained healthcare systems. While dealing with viral infections as a healthcare concern is not new, our exceptionally mobile society has added to the critical challenge of limiting pathogen spread of a highly transmissible virus prior to the generation, testing, and distribution of safe and effective vaccines. The tremendous global effort put forth to address the recent pandemic induced by SARS-CoV-2 infection has highlighted many of the strengths and weaknesses of how vaccines are identified, tested, and used to provide protection. These uncertainties are exacerbated by the lack of clear and consistent messaging that can occur when the processes of research, development, and clinical testing that normally requires years of study and consideration are compressed into a few months. In this commentary, I will provide some background on the intramuscular (IM), subcutaneous (SC), and intradermal (ID) administration routes used for injectable vaccines and some information on potential immunological outcomes. With this background, I will address the recent FDA decision to allow an approved vaccine against monkeypox virus to be administered by ID, as well as its initial approval route via SC, injection as a dose-sparing strategy to maximize immunization numbers using current stockpiles.

## Vaccinology Focuses on Getting a Safe and Beneficial Immunization Outcome

Vaccinology, or the science of vaccines, is based upon basic understandings of immunogens, the host immune response, delivery strategies and technologies, manufacturing, and clinical evaluation. It does not necessarily involve a deep understanding of why or how a vaccine works to protect individuals from a specific pathogen; that is more of an immunological question. While it is easy to appreciate that a myriad of factors, such as genetics, age, sex, immune status, and general health, can affect the effectiveness of a vaccination, it is still unclear in some instances exactly how/why a particular vaccination is or is not effective [1]. Data generated by the two intertwined, yet distinct disciplines of vaccinology or immunology clearly demonstrates that vaccinations performed at different tissues of the body can generate a distinct bias in the nature and durability of the observed immunization outcome(s), with optimal beneficial vaccine outcomes often occurring when immune mechanisms are activated at the tissue that would initially engage that pathogen. Thus, immune elements present at various sites of the body used for immunization, while having many similarities, would have some specific elements to protect from a subsequent pathogen challenge most effectively at that specific site. Further, an effective immune response to protect from a pathogen challenge must find a balance between being sufficiently aggressive to ensure pathogen destruction and modulation of this immune response to limit damage to the host to ensure survival, i.e., safety and efficacy.

So, what is an effective immune response? The answer depends upon whether the goal is to achieve true prophylaxis versus corrective immunity. A true prophylaxis would come from a standing immune function that blocks the initial infection of a pathogen at the site of challenge. This would be particularly valuable in the case of HIV, for example, where this retrovirus integrates into the DNA of immune cells of the host to disrupt their capacity to participate in an effective, corrective immune response. True prophylactic protection involves constant and focused immunological vigilance at the critical sites of potential pathogen challenge, while corrective immunity induced at one site can allow a sufficient immune response following pathogen challenge at many sites of the body (since it is the systemic correction that is important and not the local blockage of infection). Most vaccines do not need to be truly prophylactic, focusing instead on inducing a corrective immunity, i.e., the presence of an immunological memory response that rises rapidly to overwhelm and clear a pathogen at an early stage of infection. In both types of immune strategy, the vaccine is intended to teach the immune system about critical elements of the pathogen required for initial infection or replicative capacity, and this typically involves the generation of a robust memory response through the actions of immune cells and effector proteins known as cytokines. Thus, it is not uncommon for someone to experience flu-like symptoms following a vaccination caused by elevated cytokine levels that are more intense than those experienced from an actual infection.

## Practical and Historical Aspects of Injected Vaccine Routes

Since many of the most virulent pathogens infect through a mucosal (respiratory, gastrointestinal, or genitourinary tract) surface, vaccination at these sites is often the strategy to generate local prophylactic response(s). A major limitation of mucosal vaccination is the challenge of delivering non-pathogenic immunogens into the epithelial cells and/or the underlying lamina propria in a manner that emulates an actual infection by that pathogen. As one might expect, there are many physical, physiological, and biological hurdles in place at these mucosal surfaces to maintain homeostasis and make it difficult for materials the size of viruses to enter. Pathogens, however, have developed elaborate mechanisms to overcome these hurdles through their infective processes. Thus, there are severe challenges for safe and efficient immunogen delivery at mucosal surface to achieve a consistent immunization. An added issue is that mucosal immunity appears to be more dynamic than systemic immunity, likely in response to the ever-changing environmental challenges being confronted, with the likelihood of vaccination boosts being needed in the timeframe of months rather than years to maintain truly prophylactic protection.

While mucosal vaccination can also generate a corrective vaccination outcome, the complex delivery challenges associated with mucosal vaccination can be eliminated by parenteral injection, commonly achieved via the IM, SC, or ID routes. Intravenous (IV) administration can technically be used for parenteral vaccine administration, but this route is not generally used since vaccines are commonly formulated as a depot to incite a more robust and durable response, and such depots are not amenable for administration by the IV route [2]. There are several anatomical distinctions to be considered, however, between IM, SC, and ID vaccine injection routes. The ID injection site is a dense connective tissue bed that contains hair follicles, blood vessels, lymphatic vessels, and sweat glands with limited potential for expansion. By comparison, injections into the SC site enter into an expandable space composed of a loose connective tissue structure housing adipocytes. The IM injection site is also an expandable environment able to accept an injected material as it is dispersed between muscle fascicles. SC injections for vaccination are commonly from 1–1.5 mL in volume [3], IM injection are 2–5 mL [4], but 50 µL or less for ID injections [5]. Transition from an SC injection to ID typically involves reducing the injected dose volume to 10–20% of the full dose delivered either SC or IM while remaining safe and efficacious. Multiple studies comparing the full dose IM to reduced dose ID administrations of inactivated poliovirus have shown sufficiently promising results to warrant adoption of this change for both routine and supplementary vaccinations as a dose-sparing strategy [6].

ID vaccinations were probably the first intentional immunization strategy, being achieved by applying inactivated whole pathogens to skin sites damaged by physical scoring [7]. This approach allowed the host’s immune system to establish an immunity to the pathogen based upon an individual’s unique immune system repertoire. With the advent of needles and syringes, it became possible to deliver an exact quantity of a vaccine material in a reproducible manner, leading to SC or IM injections becoming a standard immunization strategy. With such standardized injections came the concept of augmenting the immune response by incorporating adjuvants as part of an injected depot [8]. The outermost layer of human skin is known as the epidermis with a thickness of 0.05–0.2 mm; beneath the epidermis is the dermis which is a network of collagen fibers only 1.5–3 mm thick with the SC space below that being quite viable in thickness. IM injections are administered at a 90º angle to the skin surface with a needle that is sufficiently long to reach a striated muscle after penetrating the epidermis, dermis, and SC tissues, while SC injections are given at a 45º angle. ID injections, however, are given and at a 10–15º angle. This very low angle, known as the Mantoux technique, can be awkward to get a needle correctly placed prior to administration [9].

## Considerations Related Changing Injection Routes for the Monkeypox Vaccine

Here is where we now should start considering potential issues associated with shifting the site of injection for a vaccine from one format to another as stated in a recent emergency FDA authorization for monkeypox immunizations (https://www.fda.gov/news-events/press-announcements/monkeypox-update-fda-authorizes-emergency-use-jynneos-vaccine-increase-vaccine-supply). The JYNNEOS vaccine was approved in 2019 to prevent monkeypox disease in adults 18 years of age and older deemed to be at high risk, being administered by SC injection in two doses, 28 days apart (https://www.fda.gov/vaccines-blood-biologics/jynneos); it contains a modified form of Vaccinia Ankara-Bavarian Nordic (MVA-BN) virus, which is a weakened, non-replicating orthopoxvirus. Due to only infrequent outbreaks around the globe, there has been limited commercial interest in the production of monkeypox vaccines, with the JYNNEOS vaccine being the only currently marketed product. A clinical study reported in 2015 demonstrated that SC injection and ID injection (one-fifth of the volume of the SC dosing material) of the JYNNEOS vaccine resulted in comparable immunological outcomes [10]. Indeed, this two-dose vaccination study showed that ID dosing, despite the reduced vaccine dose, achieved its primary objective of non-inferiority compared to the previously approved SC dosing protocol; an outcome consistent with potential immunological benefits achieved from targeting antigen-presenting and immune-competent cells present in the dermis that are present at a relatively higher density than the subcutaneous space or in striated muscle [11].

The change from SC to ID dosing for the JYNNEOS vaccine, despite this supporting clinical data, is certain to be met with public concerns regarding real-world outcomes. As a global society, we have recently experienced unprecedented skepticism and dis-information associated with the rapid development of SARS-CoV-2 vaccines to prevent COVID-19 symptoms that produce a constellation of maladies. For example, social media accounts have suggested a plethora of absurdities regarding SARS-CoV-2 vaccination including the introduction of microchips, altering a patient’s DNA, and making a person magnetic. In some ways, this is not surprising; a great deal of uncertainty can come from anything new. While the JYNNEOS vaccine is not new, very few individuals are educated about monkeypox, and even fewer would have had an ID administration in their immunization history. Thus, skepticism of the safety and efficacy of an unfamiliar vaccine given by an unfamiliar injection route should be anticipated, and this might just be the forerunner for dis-information. One can provide an immunological rational for the relative advantages of an ID immunization to protect individuals from a virus that can be transmitted by close skin-to-skin contact, but this is not what will make sense to or be perceived as important by the individual who should be submitting for a monkeypox vaccination. What will affect them the most is their perceptions of the ID vaccination experience, which translate into their real-world reality.

As most injected medicines are given by IM or SC administration, few healthcare workers have the training or experience with the Mantoux technique to deliver a proper ID injection rapidly and accurately to this thin layer of tissue beneath the epidermis and above the SC space [12]. The difficulties of achieving this outcome are further complicated when injecting a highly active child and into a modified dermis caused by age- or elasticity-related skin conditions. Indeed, if a healthcare worker fumbles with efficiently getting the needle inserted to this site or misses the site upon injection, the positive nature of the patient’s experience and possibly the desired immunological outcome could be compromised. Such real-world experiences could translate into skepticism about the vaccination process, possibly devolving into dis-information passed onto friends, relative, and beyond though social media.

## Summary

There is a rich milieu of immune-related cell types that reside in or circulate through the dermis that can generate both adaptive and innate responses: macrophages, mast cells, Langerhans cells, and dermal dendritic cells [13]. Indeed, the antigen-presenting capabilities of this repertoire of cells in the dermis can result in immune responses that are superior to other anatomical sites, with less vaccine material [14]. As ID injections are well-known for a robust immune response, this delivery strategy has been proposed for a number of vaccines, including those targeting influenza [15] and SARS-CoV-2 [16] viruses. With the promise of ID vaccination but with the challenges of efficient immunization expertise by healthcare personnel, it is not surprising that great efforts have been made to develop systems and devices to simplify and standardize these administrations [17]. Using such approaches to make ID immunization as simple as current IM or SC injections would dramatically benefit the global effort to address pathogen outbreaks to maximize the number of patients reached while minimizing the burden of vaccine manufacturing and stockpiling. Most of these approaches would also address the concern that, despite the reduced volume required for injection, the relative proportion of vaccine wastage in the dead space of the needle and syringe is increased for ID compared to the larger volumes of SC or IM injection [18].

While guidance changes issued by the FDA are frequently based upon more than a single clinical trial outcome, changing the route of the JYNNEOS vaccine from SC to an ID injection as a dose-sparing strategy is supported by robust, well-controlled clinical data and sound immunological principles. The challenge, however, will be to ensure that patients receiving these ID vaccines have a positive experience during the administration process and benefit from a properly performed injection. To successfully meet this challenge, we will need to carry out a mass immunization programs where healthcare professionals who are sufficiently trained in the Mantoux technique administer these vaccines. As ID vaccines gain acceptance by patients following positive vaccination experiences, advantages to this approach for global immunization programs will lead to clinical testing and acceptance for ID injection systems and devices. Both outcomes would hopefully limit monkeypox vaccine skepticism and dis-information like that recently seen for SARS-CoV-2 vaccines. Healthcare approaches to address the COVID-19 disease induced by the SARS-CoV-2 virus have provided a good example of how an insufficient understanding of viral pathogenicity and the requirements for mechanism(s) of immune-induced protection can result in skepticism and dis-information that suppressed vaccine acceptance by the public.
